# Mating experience and food deprivation modulate odor preference and dispersal in *Drosophila melanogaster* males

**DOI:** 10.1093/jis/14.1.131

**Published:** 2014-10-01

**Authors:** Shu-Ping Wang, Wei-Yan Guo, Shahid Arain Muhammad, Rui-Rui Chen, Li-Li Mu, Guo-Qing Li

**Affiliations:** Education Ministry Key Laboratory of Integrated Management of Crop Diseases and Pests, College of Plant Protection, Nanjing Agricultural University, Nanjing 210095, China

**Keywords:** starvation, odorant, orientation

## Abstract

Rotting fruits offer all of the known resources required for the livelihood of *Drosophila melanogaster* Meigen (Diptera: Drosophilidae). During fruit fermentation, carbohydrates and proteins are decomposed to produce volatile alcohols and amines, respectively. It is hypothesized that *D. melanogaster* adults can detect these chemical cues at a distance to identify and locate the decaying fruits. In the present paper, we compared the olfactory responses and movement of male flies varying in mating status and nutritional state to methanol, ethanol, and ammonia sources using a glass Y-tube olfactometer. In general, ethanol vapor at low to moderate concentrations repelled more hungry mated males than satiated ones. In contrast, methanol showed little difference in the attractiveness to males at different nutritional states and mating status. Moreover, ammonia attracted more hungry mated males. The attractiveness increased almost linearly with ammonia concentration from lowest to highest. When ammonia and artificial diet were put together in the odor arm, the responses of male flies to mixed odor mimicked the response to ammonia. Furthermore, odorant concentration, mating status, and nutritional state affected the flies’ dispersal. Mated and starved males dispersed at a higher rate than virgin and satiated ones. Thus, our results showed that starved, mated males increased dispersal and preferred ammonia that originated from protein.

## Introduction

In *Drosophila melanogaster* Meigen (Diptera: Drosophilidae), mating induces the female’s oogenesis and vitellogenesis and stimulates ovulation and egg deposition. During the first day after mating, a female lays up to 80 eggs. Consequently, mated females require large quantities of macronutrients, especially amino acids. In order to meet the nutritional needs, the mated females dramatically modify their physiology and behavior ( Ribeiro and Dickson 2010 ; Vargas et al. 2010 ). These modifications include increased dispersal of females when food is unavailable ( Simon et al. 2011 ), an increase in food intake ( Carvalho et al. 2006 ), preferential shift toward a high-protein diet ( Ribeiro and Dickson 2010 ; Vargas et al. 2010 ), and a higher level of general activity ( Isaac et al. 2010 ). These physiological and behavioral shifts can be enhanced by starvation ( Ribeiro and Dickson 2010 ; Vargas et al. 2010 ).

In males, the seminal fluid content in the reproductive tract declines to a low level after mating ( Duportets et al. 1998 ; Reinhardt et al. 2011 ). In some insect species, such as *Acanthoplus discoidalis* ( Bateman and Ferguson 2004 ), *Agrotis ipsilon* ( Barrozo et al. 2010a , b), *Ephippiger ephippiger* ( Ritchie et al. 1998 ), *Euborellia annulipes* ( Rankin et al. 2009 ), *Gryllus bimaculatus* ( Ureshi and Sakai 2001 ), and *Spalangia endius* ( King et al. 2005 ; Fischer and King 2008 ), there is a refractory postejaculatory interval following copulation. This interval allows newly mated males to “wait” and eventually feed to replenish their reproductive tracts. In *D. melanogaster,* five copulations in rapid succession lead to a depletion of seminal fluids ( Lefevre Jr. and Jonsson 1962 ; Linklater et al. 2007 ). Moreover, the accessory glands show a postmating increase in gene expression ( Herndon et al. 1997 ), and the production of seminal fluid is upregulated before an anticipated high mating rate ( Fedorka et al. 2011 ). Therefore, it is reasonably hypothesized that newly mated *D. melanogaster* males prefer high-protein food in order to obtain enough amino acids to rapidly synergize seminal fluid proteins to refill their reproductive tract. However, the hypothesis remains to be confirmed.

*D. melanogaster* male flies mainly rely on odors to find food ( Smith 2007 ). Moreover, mating status (virgin or mated) and nutritional state (starved or satiated) may modify olfactory responses of the males ( Becher et al. 2010 ; Simon et al. 2011 ). During fruit fermentation, carbohydrates are decomposed to produce some alcohol, aldehyde, ketone, acid, and ester volatiles ( Pfeiler and Markow 2003 ; Forbes 2008 ), whereas the breakdown of proteins generates some volatile nitrogenous compounds such as amines ( Dent et al. 2004 ). On *D. melanogaster* antennae, three types of olfactory sensory hairs (basiconic, trichoid, and coeloconic) have been found ( Benton et al. 2009 ). Of the three types of sensilla, basiconic and trichoid sensilla are generally broadly tuned to many alcohols and esters, whereas coeloconic sensilla are narrowly tuned to amines and acids ( Silbering et al. 2011 ). The distinct olfactory properties of the sensilla indicate that both alcohols and amines may be important chemical cues for the flies to assess nutritional quality of the rotting fruits.

It has also been reported that starvation enhances *D. melanogaster* dispersal ( Simon et al. 2011 ; Isaac et al. 2010 ). For example, headspace volatiles from vinegar stimulated upwind flight attraction in 62% of starved flies during a 15 min experimental period, but only attracted less than 20% of satiated flies ( Becher et al. 2010 ).

Accordingly, we hypothesize that mated *D. melanogaster* males are more likely than virgin males to disperse and orient to amine sources rather than to alcohol in order to obtain high-protein food. Moreover, the likelihood should be enhanced by starvation. Here, we selected two alcohols (methanol and ethanol) and an amine (ammonia) and compared the differences in the olfactory responses and movements between mated and virgin and starved and satiated male flies using a glass Y-tube olfactometer.

## Materials and Methods

### The flies

The *D. melanogaster* flies used in this study were Canton-S strain. The flies were raised on conventional cornmeal/sucrose/agar artificial diet (cornmeal 5%, sucrose 10.5%, yeast 2% and agar 0.7%) under controlled temperature (25 ± 1°C), photoperiod (12:12 L:D), and humidity (-50% RH). The methods for culturing flies and the cooking recipe were according to a method described by the Bloomington Drosophila Stock Center at Indiana University ( http://flystocks.bio.indiana.edu ). Pupae were sexed on the basis of presence/absence of male sex combs ( Zhong et al. 2010 ).

### Animal handling

Unless otherwise noted, we housed 30 newly emerged flies in a rearing vial (3 cm in diameter and 12 cm in height) containing a 0.5 × 0.5 × 0.5 cm block of standard cornmeal-sucrose-yeast agar diet for a period of three days.

In order to compare mated and virgin flies of a similar age and rearing condition, we collected virgins less than 6 hr post-eclosion and divided the collected individuals into two groups: 30 males per vial, and a mixture of 15 males and 15 females per vial. To keep housing densities equivalent, three days later we combined vials containing the mixture of 15 males and 15 females (providing ample time for them to mate) and then sorted them by sex into two new vials. Both the virgin and mated males were separated into two groups: the first was transferred into new vials with the artificial diet, and the second was housed in vials containing only agar in order to deprive the flies of food but not water. The following day, we tested these virgin and mated males. To facilitate counting and sorting, we anesthetized the flies in rearing vials with a pulse of CO2.

### Chemicals and preparation of test solutions

Methanol and ethanol were purchased from Nanjing Chemical Reagent Co., Ltd., China. Ammonium bicarbonate (NH4HCO3) was acquired from Shanghai Sangon Biological Engineering Technology and Services Co., Ltd., China. All chemicals were analytical grade. These three chemicals were individually dissolved and diluted with distilled water to obtain seven to nine solutions, with final concentrations (wt./vol.) of 0.25%, 0.5%, 1.0%, 2.0%, 4.0%, 8.0%, 16.0%, 32.0%, and 64.0% for methanol and ethanol, and 0.25%, 0.5%, 1.0%, 2.0%, 4.0%, 8.0%, and 16.0% for ammonium bicarbonate.

### Bioassay

To assay the behavioral response of males to the rotting fruit-borne volatile odorants methanol, ethanol, and ammonia, a glass Y-tube olfactometer similar to that reported by Fuyama (1976 , 1978) was used. The olfactometer consisted of two major parts: a starting tube 22 cm long and 3.6 cm inside diameter, and two choice arms 18 cm long and 3.6 cm inside diameter. The Y-tube olfactometer was placed horizontally in a dark room at 25ºC and covered with black cloth in order to avoid any visual stimuli.

An outline of the air delivery system is as follows: air from outside is compressed with a gas compressor and cleaned by flowing through an active charcoal column; then the flow rate is regulated using a flowmeter and a needle valve to provide 20 L/hr airflow. Thereafter, the airflow is bubbled into a 300 mL gas washing bottle containing 100 mL of distilled water to attain a constant vapor pressure and is divided by a Y-connector and led into two choice tubes. All parts of the air delivery system consist of glass and Teflon tube.

Just before the bioassay, an aliquot of 100 μL of test solution (treatment) or distilled water (control) was applied to a piece of filter paper (1 cm in diameter). Immediately after application, a pair of filter papers (a treatment and a control) were respectively inserted into one of the two choice arms of the Y-tube olfactometer to supply odor or as a control. In order to mimic natural odorants, we also put ammonia-treated paper and 1 gram of artificial diet in the same odor arm of the Y-tube olfactometer. The allocation of odor and control arm was changed from experiment to experiment to eliminate possible right-left bias.

Y-tube olfactometer tests were carried out with a single individual in some species (Steiner and Ruther 2009; Louly et al. 2010 ), but with a group of individuals in other species ( Fuyama 1976 , 1978; Lefèvre et al. 2010 ). Our preliminary tests showed that the test results were similar no matter whether a single male or a group of males was used. To simplify the experiment, 30 male flies were introduced into the starting tube in each test, and then the gas compressor was opened to allow airflow into the olfactometer. The flies were allowed to run and fly freely for 30 min.

At the end of the free period, the number of flies in each choice tube and in the starting tube was counted. After each run, the flies were discarded and the olfactometer was washed with distilled water and dried in open air in order to eliminate the interference of residue odors. All tests were replicated five times, with completely naïve flies used for each test and each replicate. The mean of five independent groups of each concentration was used to assess the response of flies to the three odorants.

The response was evaluated by an index designated as “preference index” (PI), calculated as follows: PI = (number of flies which entered the odor arm **—** number of flies which entered the control arm) / (number of flies which entered the odor arm + number of flies which entered the control arm). The index theoretically varies between -1 (extreme repellency) and +1 (extreme attractiveness).

Moreover, it has been reported that the number of unresponsive flies (which remain in the starting tube at the end of the experiments) reveals movement differences to different odor sources ( Fuyama 1976 ). In the present paper, therefore, the number of unresponsive flies was used to compare dispersal behavior of males.

### Statistical analysis

The data were given as mean ± SE and were analyzed by ANOVA followed by the Tukey-Kramer test, using SPSS 16.0 for Windows (IBM, www.ibm.com ).

## Results

### Responses to odorants

When the results of the same odorant were pooled, the preference indices of males to ethanol, methanol, and ammonia did not exhibit significant differences ( *P* > 0.05, one-way ANOVA). Therefore, ANOVA analyses were performed for each odorant ( Table 1 ). Statistically significant differences were found among tested concentrations, between mated and unmated flies, and between nutritional states. The interactions between concentration and mating status, concentration and nutritional state, mating status and nutritional state, and among concentration, mating status, and nutritional state were not statistically significant ( Tables 1 ).

**Table 1.**
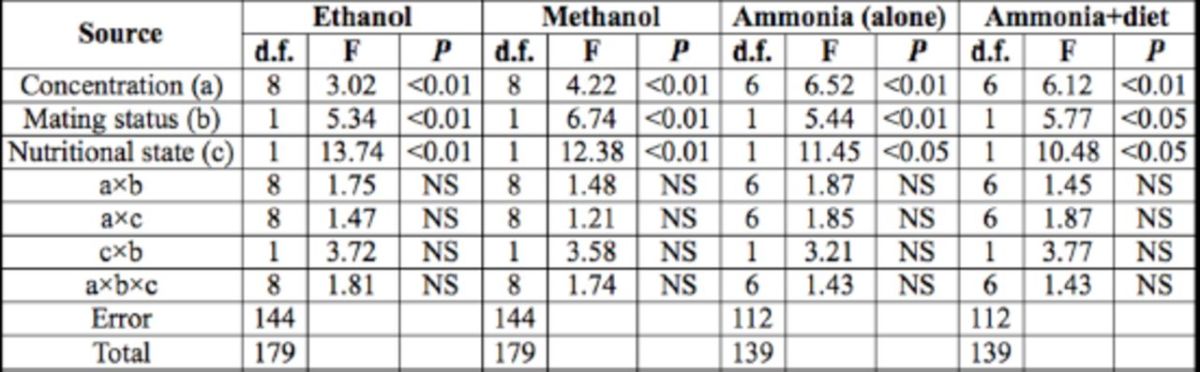
ANOVAs for the preference indices of *D. melanogaster* male flies to three chemical odorants.

Above statistical computations were performed by the program SPSS 16.0 for Windows. See text for further details. NS = not significant.

### Responses to alcohol odors

**Figure 1.**
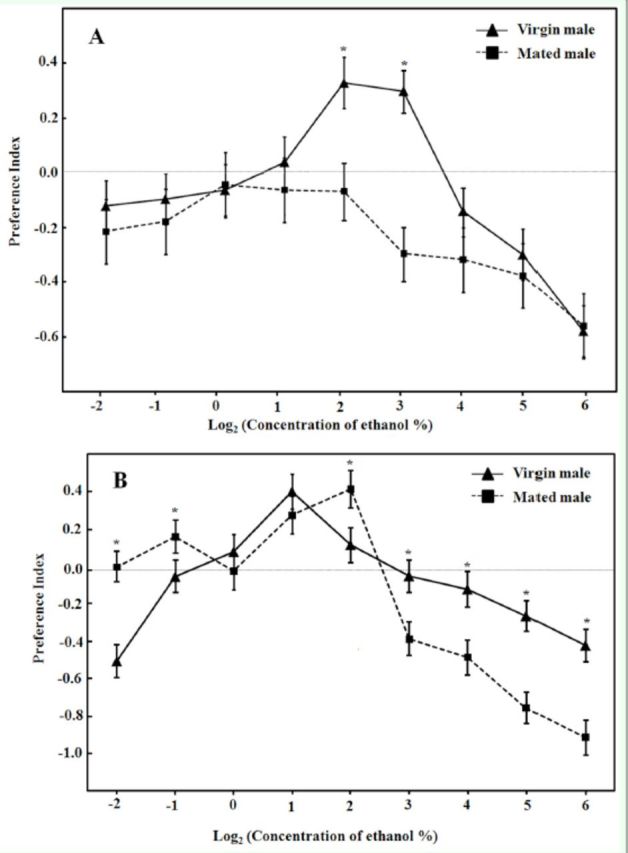
Dose-response curves for ethanol of *D. melanogaster* mated and virgin males with (A) or without (B) food deprivation prior to experiment. Each point represents the average of four replicates. PI = (number of flies which entered the odor arm - number of flies which entered the control arm)/(number of flies which entered the odor arm + number of flies which entered the control arm). * indicates significant differences between virgin and mated males at *P* < 0.05 by .AN OVA.

**Figure 2.**
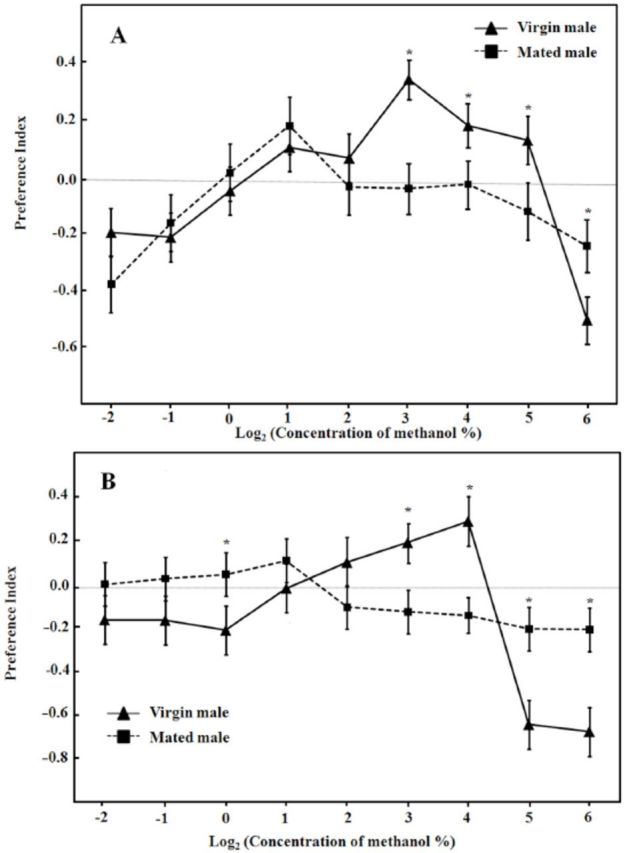
Dose-response curves for methanol of *D. melanogaster* mated and virgin males with (A) or without (B) food deprivation prior to experiment. Each point represents the average of four replicates. PI = (number of flies which entered the odor arm - number of flies which entered the control arm)/(number of flies which entered the odor arm + number of flies which entered the control arm). * indicates significant differences between virgin and mated males at *P* < 0.05 by ANOVA.

As seen in Figures 1 and 2 , the adult males showed a typical hormetic response to the two alcohols, i.e., a nonlinear biphasic dose– response relationship characterized by small quantities having opposite effects from large quantities ( Calabrese and Baldwin 2003 ). For ethanol, the preference indices increased gradually with concentration from an approximate threshold of 0.25% (-2 in log scale; hereafter the log-scale concentration will be shown in parentheses after the percentage) to maximum responses at 2% (1) or 4% (2), after which they abruptly decreased toward repulsion; the highest repulsion was shown at the concentration of 64% (6) or 0.25% (-2). For methanol, the preference indices rose gradually from the concentration of 0.25% (-2) to maximum responses at 2% (1) or 8% (3), after which they dropped toward repulsion; the highest repulsion was seen at the concentration of 64% (6) or 0.25% (-2) ( Figures 1 and 2 ).

Mating status and food deprivation affected the attractiveness of the two alcohols ( Table 1 ). In general, ethanol vapor at low and middle concentrations repelled more hungry mated males than satiated ones. In contrast, methanol showed little difference in the attraction of males at different nutritional states and mating status ( Figures 1 and 2 ).

### Responses to ammonia odor

**Figure 3.**
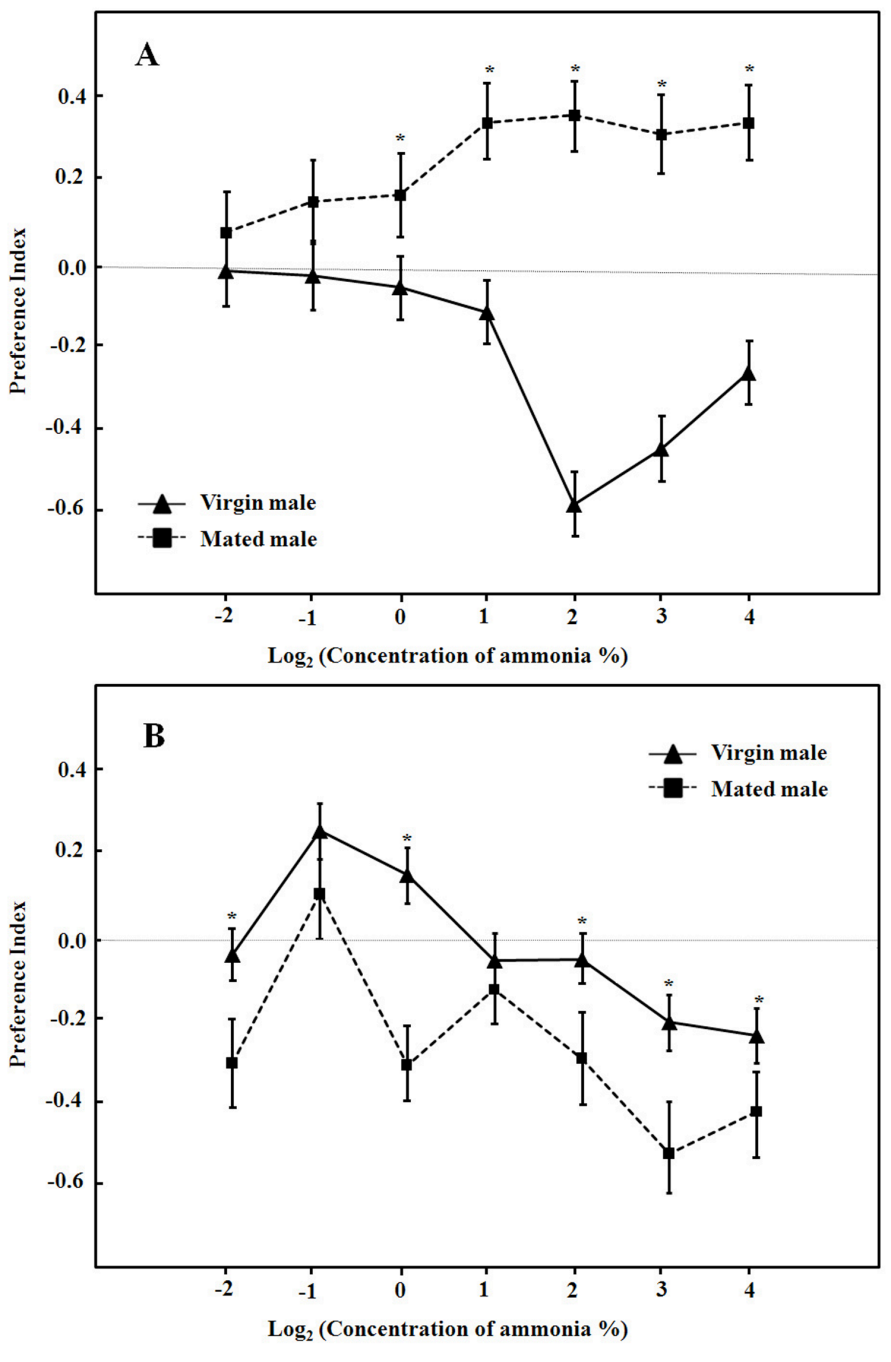
Dose-response curves for ammonia (alone) of *D. melanogaster* mated and virgin males with (A) or without (B) food deprivation prior to experiment. Each point represents the average of four replicates. PI = (number of flies which entered the odor arm - number of flies which entered the control arm)/(number of flies which entered the odor arm + number of flies which entered the control arm). * indicates significant differences between virgin and mated males at *P* < 0.05 by ANOVA.

Satiated males showed a typical hormetic response to ammonia. The preference indices increased with concentration from 0.25% (-2) to maximum response at 0.5% (-1), after which they lowered toward repulsion; the highest repulsion was found at the concentration of 16% (4). At all the tested concentra-concentrations, virgin males exhibited higher preferences than mated ones ( Figure 3B ).

Food deprivation changed response pattern to ammonia ( Table 1 ). For mated males, the preference indices rose almost linearly with concentration from the lowest to the highest, with maximum responses seen at 16% (4). For virgin males, the preference indices dropped when the concentration increased. Moreover, ammonia was attractive to hungry mated males and repellent to starved virgin ones at the concentrations of 2%, 4%, 8%, and 16% ( Figure 3A ).

In order to mimic natural odorants, we put ammonia and artificial diet in the same odor arm of the Y-tube olfactometer and examined the preference indices ( Table 1 , Figure 4A, B). In general, the responses of male flies to mixed odors resembled those to ammonia.

### Dispersal differences of male flies

When the results of the same odorant were pooled, the average number of unresponsive flies to ethanol, methanol, and ammonia were not significantly different ( *P* > 0.05, one-way ANOVA). Therefore, ANOVA analyses were performed for each odorant ( Tables 2 and 3 ).

**Table 2.**
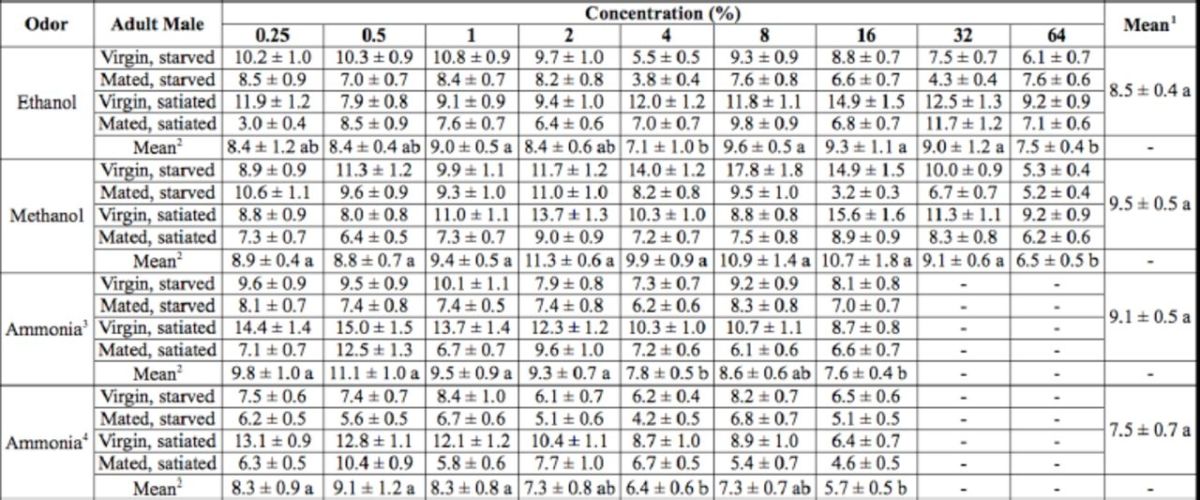
The numbers of unresponsive *D. melanogaster* male flies to three chemical odorants at different concentrations.

The data were given as means ± SE. 1 are the average numbers of unresponsive flies to ethanol, methanol, or ammonia, respectively, at all tested concentrations. 2 are the average number of unresponsive flies to ethanol, methanol, or ammonia, respectively, at each of the tested concentrations. 3 and 4 mean ammonia used alone or combined with diet. The data were subjected to one-way ANOVA followed by the Tukey–Kramer test. Means followed by the same letter (a and b) are not significantly different at *P* < 0.05.

**Table 3.**
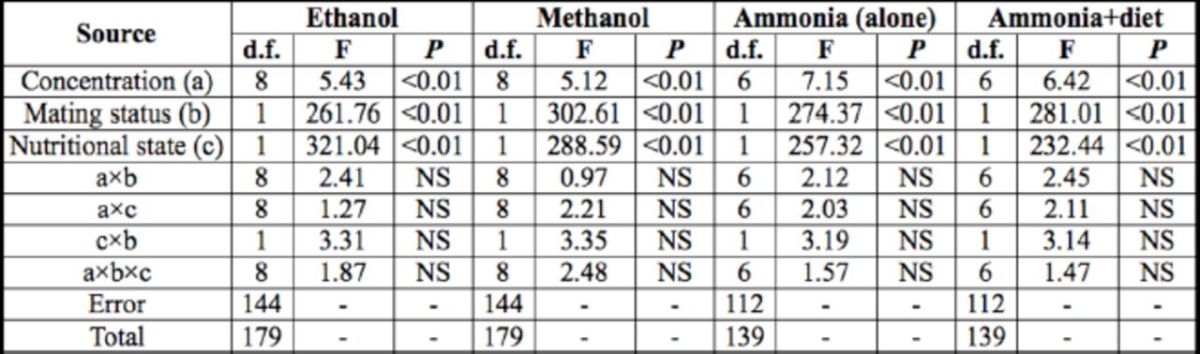
ANOVAs for the numbers of unresponsive *D. melanogaster* male flies to three chemical odorants.

Above statistical computations were performed by the program SPSS 16.0 for Windows. See text for further details. NS = not significant.

Significant differences were found among tested concentrations, between mated and un-mated flies, and between nutritional states. The interactions between concentration and mating status, concentration and nutritional state, mating status and nutritional state, and among concentration, mating status and nutritional state were not significant ( Tables 2 and 3 ).

Mating status significantly affected the numbers of unresponsive male flies. When the results of all odorant-specific concentrations were pooled, the average numbers of virgin and mated males unresponsive to ethanol were 9.8 ± 0.4 and 7.2 ± 0.5, respectively; to methanol were 11.1 ± 0.5 and 7.9 ± 0.3, respectively; to ammonia were 10.5 ± 0.5 and 7.7 ± 0.3, respectively; and to ammonia plus diet were 8.8 ± 0.6 and 6.2 ± 0.4, respectively. Food deprivation also changed the numbers of unresponsive male flies. Summing up all unresponsive male flies at all tested concentrations, the average values in starved and satiated groups to ethanol were 7.8 ± 0.4 and 9.3 ± 0.4, respectively; to methanol were 9.1 ± 0.3 and 9.8 ± 0.4, respectively; to ammonia were 8.1 ± 0.5 and 10.1 ± 0.3, respectively; and to ammonia plus diet were 6.4 ± 0.5 and 8.0 ± 0.7, respectively ( Tables 2 and 3 ). It seems that mated males dispersed at a higher rate than unmated ones in response to food-originated odors. Similarly, hungry males were more likely to disperse compared to satiated ones.

## Discussion

Rotting fruits offer all of the known resources required for the livelihood of *D. melanogaster.* Therefore, it is important for *D. melanogaster* males to rapidly find decaying fruits for food and potential mates. To effectively perform this task, the males are mainly dependent on volatile odorants to identify and locate rotting fruits ( Smith 2007 ). The key volatile odorants can be connected either directly to the fruit or to microorganisms living in and on the fruit ( de Bruyne et al. 2001 ; Stensmyr et al. 2003 ; Hansson et al. 2010 ; Becher et al. 2012 ). Carbohydrates are decomposed to produce alcohol, aldehyde, ketone, acid, and ester volatiles ( Pfeiler and Markow 2003 ; Forbes 2008 ); among the products, ethanol, acetic acid, acetoin, 2-phenyl ethanol, and 3-methyl-1-butanol (but not methanol) can mimic fermenting fruits to attract flies ( Becher et al. 2012 ). Moreover, the breakdown of proteins generates some volatile nitrogenous compounds, such as amines ( Dent et al. 2004 ). Of the amines, ammonia can attract many Drosophilid species ( Thomas 2003 ; Leblanc et al. 2010 ).

In our comparison of the olfactory responses and movement of male flies varying in mating status and nutritional state to methanol, ethanol, and ammonia sources using a glass Y-tube olfactometer, ethanol and methanol vapors generally did not attract more hungry mated males than satiated virgin ones. In contrast, ammonia attracted more hungry mated males. The attractiveness increased almost linearly with ammonia concentration from the lowest to the highest. When ammonia and artificial diet were put together in the same odor arm, the responses of male flies to mixed odor resembled those to ammonia. Furthermore, mated and starved males dispersed at a higher rate than virgin and satiated ones in the presence of the odorants originating from decaying fruits. Thus, our results showed that starved mated males increased dispersal and preferred ammonia that originated from protein.

Similarly, Becher et al. (2010) have reported that flies exhibit different orientation and flight to fruit-derived odorants depending on their mating status (virgin or mated) or nutritional state (starved or satiated). Moreover, ammonia is highly attractive to many insect species in Diptera, Lepidoptera, Hymenoptera, Coleoptera, Hemiptera, Neuroptera, and Orthoptera ( Thomas 2003 ; Shen et al. 2009 ; Leblanc et al. 2010 ; Yu et al. 2011 ). These insects may associate ammonia, through olfactory receptors, with high-protein foods, animal hosts, or other nutritional resources ( Mazor et al. 1987 ; Kendra et al. 2005 ; Manrakhan and Lux 2008; Shen et al. 2009 ; Yu et al. 2011 ).

Moreover, the movement of flies is also affected by various fruit-derived odorants. For example, Simon et al. (2011) have reported that mated or hungry flies disperse at a higher rate. Moreover, vinegar, a food-borne volatile, stimulated upwind flight in hungry *D. melanogaster* males, although mating status had little effect on the rate of attraction ( Becher et al. 2010 ). The discrepancy of our results and those reported by Becher et al. (2010) may result from different chemicals and/or different experiment apparatus.

In summary, our results revealed that starved mated males increased dispersal and preferred the volatile chemical that originated from protein. Similarly, mating also changed olfaction in several other insect species ( Jang et al. 1998 ; Cornelius et al. 2000 ; Reddy and Guerrero 2000 ; Barrozo et al. 2010a , c; Barrozo et al. 2011 ). Whether mated *D. melanogaster* males were similar to their female mates and shifted their preference to a high-protein diet ( Ribeiro and Dickson 2010 ; Vargas et al. 2010 ) will require food choice experiments to confirm.
